# Recent advances in searching c-Myc transcriptional cofactors during tumorigenesis

**DOI:** 10.1186/s13046-018-0912-2

**Published:** 2018-09-27

**Authors:** Matteo Caforio, Cristina Sorino, Stefano Iacovelli, Maurizio Fanciulli, Franco Locatelli, Valentina Folgiero

**Affiliations:** 1 Department of Pediatric Hematology/Oncology and of Cell and Gene Therapy, Bambino Gesù Children’s Hospital, 00146 Rome, Italy; 2 SAFU, Department of Research, Advanced Diagnostics, and Technological Innovation, Translational Research Area, Regina Elena National Cancer Institute, 00144 Rome, Italy; 3 Department of Pediatric Science, University of Pavia, 27100 Pavia, Italy

**Keywords:** c-Myc, Transcription cofactors, Tumorigenesis

## Abstract

**Background:**

The mechanism by which c-Myc exerts its oncogenic functions is not completely clear and different hypotheses are still under investigation. The knowledge of the capacity of c-Myc to bind exclusively E-box sequences determined the discrepancy between, on the one hand, genomic studies showing the binding of c-Myc to all active promoters and, on the other hand, the evidence that only 60% or less of the binding sites have E-box sequences.

**Main body:**

In this review, we provide support to the hypothesis that the cooperation of c-Myc with transcriptional cofactors mediates c-Myc-induced cellular functions. We produce evidence that recently identified cofactors are involved in c-Myc control of survival mechanisms of cancer cells.

**Conclusion:**

The identification of new c-Myc cofactors could favor the development of therapeutic strategies able to compensate the difficulty of targeting c-Myc.

## Background

Myc is the most frequent amplified oncogene in human cancers and its alteration is observed in a wide range of tumors, including breast, lung and prostate cancer [1]. Deregulated expression of Myc in cancers occurs through gene amplification, chromosomal translocation, focal enhancer amplification, germline enhancer polymorphism or, commonly, through constitutive activation of upstream signaling pathways [2]. The link between Myc and cancer was greatly strengthened by the discovery that avian leukosis virus (ALV)-induced B-cell lymphomas consistently contained retroviral insertions in the vicinity of the Myc gene [3]. This means that the oncogenic properties of Myc are not only manifested by the retroviral-transduced v-myc, but can also occur as a consequence of viral perturbation of cellular Myc. It was also clear that Myc can be complicit in neoplasms that lack any retroviral involvement [4]. In 1985, Adams et al. demonstrated that Myc is crucial for the genesis of B-cell lymphomas through the generation of transgenic mice carrying an Ig-enhancer linked to Myc, this consolidating the notion of a strong involvement of Myc in hematological tumors [5]. Thus, all three vertebrate Myc family members (c-Myc, MYCN and MYCL1) are involved in the etiology of human cancers [4]. c-Myc is a rapidly degraded protein with half-life of 20-30 minutes [6] and a variety of different proteins interact with c-Myc to control its stability and transcriptional activity. The oncogenic potential of c-Myc stems from its function as transcriptional regulator that binds DNA on heterodimerization with myc-associated factor X (MAX) [7]. The carboxyl terminus of c-Myc encodes a 100-residue basic helix-loop-helix-leucine-zipper (bHLH-LZ) DNA-binding domain. The leucine zipper forms a coiled-coil heterodimer with a homologous region on the transcriptional repressor MAX, which together engage E-box DNA-binding sites [4]. Localization of the heterodimer to either promoter or enhancer regions positively regulates transcription of proliferation-associated genes through control of transcription elongation [8]. In addition to its canonical function as transcriptional activator, c-Myc induction causes transcriptional repression of target genes [9]. The discordance in c-Myc-dependent genomic binding and expression analysis suggests that target gene expression after binding to DNA is highly regulated by the presence of specific cofactors. Indeed structural studies indicate that the Myc-MAX dimeric region presents a large solvent-accessible surface area forming a platform for binding by other factors [2]. These can act as molecular switches to mediate c-Myc-induced proliferation and tumorigenesis, suggesting that dynamic complexes of cofactors can differentially regulate the transcriptional activity and target gene selection of c-Myc to mediate diverse biological outcomes [10, 11]. The sequence DNA-binding of c-Myc is specific for E-boxes and can occur only following recognition of open chromatin context. When overexpressed, the level of c-Myc that is bound to E-boxes-containing promoters increases, with more promoters becoming occupied, and c-Myc starts binding larger numbers of distal sites [10]. On the other hand, promoters of repressed genes are poorly enriched in E-boxes, suggesting that other factors recruit c-Myc to those promoters, including the molecular complex deriving from dimerization with MAX [12, 13]. Among c-Myc-induced genes, the functional categories that recur most consistently in independent studies are cell growth, cell cycle control, energy production, anabolic metabolism and DNA replication [14]. The mechanism of action of c-Myc is still not clear and two hypothesis are still competing. One proposes a model in which c-Myc functions as a direct activator or amplifier of transcription at all active loci [10]. In an alternative scenario, c-Myc activates and represses selected target genes, with RNA amplification occurring only as secondary consequence [15].

Regardless of its specific mechanism of action, c-Myc remains one of the targets for effective antineoplastic therapy, due to its deregulation in numerous tumors. Unfortunately, c-Myc presents specific, significant obstacles to develop a strategy for its direct inhibition. Indeed, c-Myc lacks enzymatic activity, this limiting those approaches that require its direct inhibition. Rather, c-Myc activity is exerted by protein-protein interactions, which remains a technical barrier impeding organized efforts in drug discovery. The biological behavior of c-Myc in physiology and disease must still be fully elucidated, requiring comprehensive mapping of its target genes and the importance of c-Myc cofactors. These molecules function, at least in part, by affecting chromatin structure through their intrinsic enzymatic activities, including ATPase/helicases, histone acetyl-transferase (HATS) and histone deacetylase (HDACs). Therefore, a possible model of targeting c-Myc could involve the inhibition of these coactivator proteins, critical to c-Myc-specific initiation and elongation.

One of the first c-Myc cofactors was discovered by Peukert K et al in 1997. The authors identificated a protein that interacts with the carboxy-terminal HLH domain of Myc, Miz-1 (Myc-interacting Zn finger protein-1). It belongs to the BTB/POZ family of zinc finger proteins and interacts with DNA in a sequence-specific manner. Both Max and Miz-1 interact with the HLH domain of Myc suggesting that Max and Miz-1 may form alternate complexes with Myc. In particular Miz-1 is involved in the c-Myc-dependent mechanism of repression of particular genes like Cyclin D1 [16]. In addition, only recently it has been demonstrated that the interaction of Myc with Miz1 is critical for the development of G3 MBs (Medulloblastoma) and distinguishes G3 from other MB subgroups [17].

McMahon et al, in 1998, showed that inhibition of TRRAP synthesis or function blocks c-Myc-mediated oncogenic activity. TRRAP with TIP49 and TIP48 is involved in chromatin modifying complexes. In particular, ATPase/helicase motifs contained in TIP49 and TIP48, when mutated, create a dominant inhibitor of c-Myc oncogenic activity [18]. Subsequently, the co-activator CBP was identified as a novel c-Myc interaction partner. These findings showed that CBP interacts directly with c-Myc and stimulates its function. Furthermore, in association with p300, CBP is recruited to c-Myc-regulated genes [19]. Fujii M et al. in 2006 demonstrated that SNIP1 functions as a regulator of c-Myc activity and that it enhances the transcriptional activity of c-Myc both stabilizing it against proteasomal degradation and bridging the c-Myc/p300 complex [20]. Then, a new model was proposed, where, in a direct feedback mechanism, ARF binds with c-Myc to inhibit canonical c-Myc target gene induction and proliferation, while inducing non-canonical expression of Egr1 and EGR1-mediated apoptosis [21]. The heterodimerization with Max is also necessary for c-Myc to recruit pTEFb, the positive transcription factor that phosphorylates the carboxy-terminal domain of RNA polymerase II, at target genes [22]. Furthermore, it is known that c-Myc requires SP1 in order to participate in the regulation of survivin promoter in controlling tumor drug resistance [23].

Recently, numerous additional c-Myc interactors have been described, further characterizing the functions of this protein and suggesting possible new therapeutic targets. In this review, we update these more recent findings about c-Myc cofactors active in tumorigenesis, with the aim to develop, through the comparison of their mechanisms of action, either novel therapy strategies or identification of selective biomarkers for diagnosis.

## Main Text

### Che-1/AATF cooperates with c-Myc in the control of BCP-ALL blast-cell proliferation

**Fig. 1:**
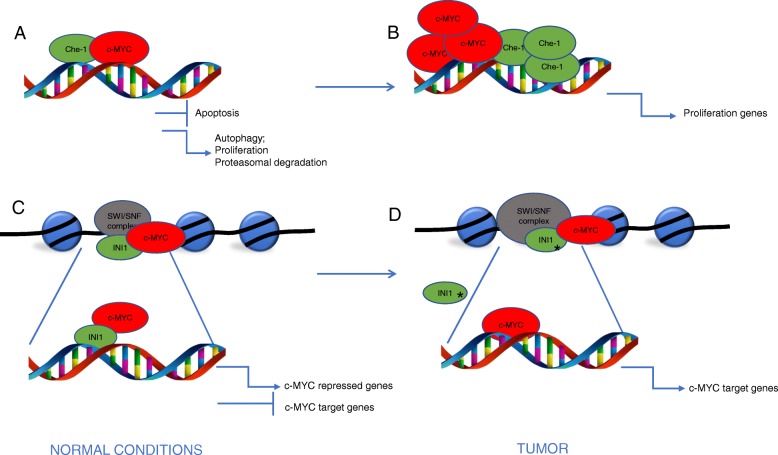
Cooperation between c-Myc and Che-1 or INI1 cofactor. **a** In physiological conditions, Che-1 exerts its function of RNA polimerase II binding protein in controlling autophagy, proliferation and proteasomal degradation and apoptosis (**b**) In tumor context (e.g. BCP-ALL), Che-1 functions as c-Myc cofactor in induction of blast cell proliferation. **c** In physiological conditions, INI1, a member of SWI/SNF complex, may regulate c-Myc transcriptional activity preventing binding to c-Myc target genes and favoring the transcription of the genes repressed by c-Myc. **d** In specific tumor context (i.e. rhabdoid cells), INI1 mutation (*) allows the transcription of c-Myc-regulated target genes

Che-1/AATF (Che-1) is a transcriptional cofactor involved in the regulation of gene expression by connecting specific transcription factors to the general transcriptional machinery. It is an ubiquitarious RNA polymerase II-binding protein exerting many cellular functions in diverse solid tumors. Che-1 promotes cell cycle progression by inhibiting the growth suppression functions of the pRb protein [24], and by controlling mitotic entry through its localization at interphase centrosomes, where it directs centrosome duplication and spindle formation [25]. Che-1 anti-apoptotic activity is exerted through its ability to counteract NRAGE-induced apoptosis. Indeed, NRAGE overexpression induces Che-1 degradation by targeting it to the ubiquitin-proteasome pathway [26]. Upon DNA damage, Che-1 is phosphorylated by checkpoint kinase MK2 inducing translocation from the cytoplasm to the nucleus, where Che-1 inhibits transcription of p53-dependent pro apoptotic genes [27]. When the DNA damage is too severe and cannot be repaired, Che-1 is degraded to execute the apoptotic program [28]. On the other hand, Che-1 is required for the transcription of the mutant forms of p53 and, in these tumor contexts, Che-1 depletion induces apoptosis through the activity of p73. In addition to these consolidated roles, it has been demonstrated that Che-1, through the inhibition of mTOR, is able to induce autophagy, allowing cells to survive under metabolic stress [29]. In addition, it has been demonstrated the involvement of Che-1 in cell metabolic adaptation upon hypoxic conditions where Che-1 depletion leads to reduction of glucose and glutamine consumption, associated with reduced inhibition of oxygen consumption and with a decreased activation of glycolytic enzymes [30]. The role of Che-1 in pediatric hematological tumors has only recently been investigated. In particular, in B-cell precursor acute lymphoblastic leukemia (BCP-ALL), Che-1 is required for control of the expression of several genes involved in cell growth, as demonstrated by ChiP-seq assay showing the presence of Che-1 on 2,205-derived TSSs, including promoters of cell cycle regulatory genes. Since c-Myc was found associated with high risk of relapse in BCP-ALL, its possible relation with Che-1 was investigated. It was demonstrated that in blast cells collected from BCP-ALL patients c-Myc binds Che-1 promoters and the two molecules were overexpressed both at the onset and at time of relapse of the disease. In-depth bioinformatic studies revealed that Che-1 and c-Myc regulate the expression of the same genes in BCP-ALL cells, preferentially involved in the control of cell proliferation. In addition Che-1 down-regulation produced a strong reduction in c-Myc recruitment on cell cycle gene promoters. Ectopic expression of Che-1 was able to counteract the effect of c-Myc depletion, this supporting the role of Che-1 as c-Myc cofactor in controlling proliferation of blast cells in BCP-ALL [31] (Fig. 1a, b).

### INI1/SNF5 interacts with c-MYC to inhibit the expression of its target genes

INI1 is a protein that belongs to SWI/SNF complex, an important chromatin remodeler. This complex is pivotal for transcriptional activity, because it allows transcriptional machinery to sit on DNA, freeing the chromatin structure from nucleosomes. SWI/SNF is considered a tumor suppressor and results either deregulated or mutated in many types of tumor [32]. INI1 is a core protein of this complex and is encoded by SMARCB1 gene. When it lacks, SWI/SNF complex is not able to assemble itself. The only deregulation of INI1 is sufficient to develop a tumor and for this reason INI1 is considered a tumor suppressor. An important example is the pediatric malignant rhabdoid tumor, in which INI1 results deregulated [33]. During the last years, the interaction between INI1 and c-Myc was initially demonstrated in HEK293T, a human kidney cell line [34]. In cancer, this interaction was demonstrated through Co-Immunoprecipitation (Co-IP) experiments in two breast cancer cell lines (SK-BR3, T47D) and two lung cancer cell lines (A549, NCI H520), and it depends on specific domains of these proteins [35]. In particular, c-Myc interacts with INI1 through the bHLHLZ region that contains leucin zipper domain. c-Myc interacts with MAX through the same domain; nevertheless, it may interact with INI1 and MAX together. Furthermore, the interaction c-Myc-INI1 is not dependent on the presence of MAX. INI1 maps its binding regions to the SNF5 homology domain, which contains two repeat regions (Rpt I, RPt II), that are amino acid sequences with ability to interact with other proteins [36]. ChIP-seq analysis indicated that there are 3279 genes that may be regulated by INI1 and c-Myc. In particular, some experiments in human rhabdoid tumor cell line (G401) showed that the presence of INI1 on the promoter of c-Myc target genes, reduces their expression. G401 is a cancer cell line that lacks detectable INI1 protein expression [37]; for this reason, it is the best cancer model for the study of the mechanism of action of INI1. When exogenous INI1 is introduced in these cells, c-Myc loses the ability to bind the promoter of its target genes. Furthermore, the introduction of INI1 in these cells increases the expression levels of c-Myc-repressed genes and decreases the expression levels of c-Myc-activated genes. This mechanism of repression is opposite to that exerted by c-Myc in breast cancer context. It has been recently demonstrated that c-Myc represses RNA-binding protein 38 (RBM38) expression through the direct binding of E-box sequences on its promoter [38]. These data indicate that INI1, when interacts with c-Myc, has an opposite behavior than c-Myc on the c-Myc-related genes. Along with the genes that are repressed by INI1, there are related cell cycle genes, indicating that INI1 acts like a tumor suppressor. Furthermore, when INI1 is overexpressed, the protein levels of c-Myc decrease, but not its mRNA expression. This finding could suggest a post-transcriptional control of c-Myc by INI1. Other studies confirmed the tumor suppressor role of INI1 and its opposite role to c-Myc, because they showed that INI1 overexpression in G401 reduces proliferation. Recent data speculate about the presence of other factors in this process [39]. In fact, other interactions on the INI1-cMyc-MAX network were demonstrated. In particular, there are other chromatin remodelers that can interact with c-Myc; for example, Nua4 and STAGA histone acetyl transferase complexes (HATs), Sin3 and Nurd histone deacetylase complexes, as well as other members of SWI/SNF complex, may belong to this network. However, INI1 is the pivotal factor of this network, because, through its interaction with c-Myc, it controls many important cell cycle genes like cyclin D1, p16 and p21, and its absence can contribute to cancer progression [40] (Fig. 1c, d).

### BPTF is a cofactor necessary for c-Myc-induced remodeling of target chromatin

**Fig. 2:**
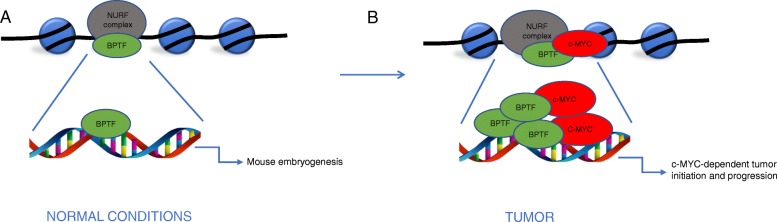
c-Myc involvement in BTPF machanism of action. **a** BPTF, a member of NURF complex, regulates genes essential for development of tissues of early murine embryogenesis (**b**) BPTF overexpression supports c-Myc in the transcription of genes involved in tumor initiation and progression.

Bromodomain PHD transcription factor (BPTF) is a subunit of mammalian NURF (ATP-dependent nucleosome-remodeling factor) that uses ATP hydrolysis to catalyse nucleosome sliding [41]. BTPF regulates genes and signaling pathways essential for the development of key tissues of the early mouse embryo [42]. Investigation on its role in cancer showed that BTPF is overexpressed in lung cancer, where it plays an essential role in cell growth and survival by targeting many signaling pathways [43]. In addition, it has been demonstrated that NURF suppresses tumor antigenicity and that its depletion improves antigen processing enhancing T-cell-mediated antitumor immunity [44, 45]. BPTF is mutated in bladder tumors and its knockdown in cultured bladder-cancer cells results in reduced proliferation and it is hypothesized that this effect is mediated in part by c-Myc [46]. In BPTF-silenced cells, an impaired activation of five independent c-Myc signatures analyzed by RNA-seq was documented. BPTF recognizes histone marks present in both high- and low-affinity c-Myc target promoters and is involved in chromatin remodeling. Indeed, in Co-IP experiments, c-Myc resulted associated with BPTF, explaining, mechanistically, the suppression in c-Myc transcription following BPTF deletion. ChIP-seq assay also revealed that BPTF regulates c-Myc binding to DNA, since BPTF silencing affects a subset of c-Myc ChIP-seq peaks. Attenuation of the c-Myc transcriptional response resulting from BPTF knockdown is associated with changes in DNA accessibility, suggesting that BPTF is necessary for the c-Myc-induced remodeling of target chromatin. Investigation of the involvement of BPTF in c-Myc-dependent biological functions revealed that BPTF deletion resulted in significantly delayed progression through S-phase and, indirectly, in a robust apoptotic response. In Burkitt’s lymphoma, colorectal, prostate and pancreatic carcinoma BTPF expression levels positively correlated with c-Myc signature, as shown by the analysis of public omics data set. Studies in *in-vivo* models showed that elimination of a single Btpf allele is sufficient to delay tumor initiation and progression. Thus, disruption of the BPTF-c-Myc interaction may represent a valuable strategy for the therapy of c-Myc-driven tumors [7] (Fig. 2a, b).

### YAP drives c-Myc transcriptional activity

**Fig. 3:**
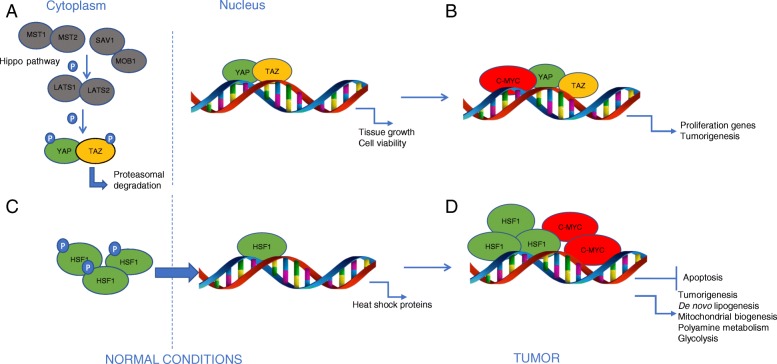
Cooperation between c-Myc and YAP/TAZ or HSF1 cofactor (**a**) Phosphorilation of YAP/TAZ by Hippo signaling pathway promotes cytoplasmic localization and ubiquitin-mediated degradation. Unphosphorilated YAP/TAZ translocate in the nucleus where they control tissue growth and cell viability (**b**) Nuclear localizazion of YAP/TAZ was observed in several tumors, where they cooperate with c-Myc in the induction of tumorigenic pathways (**c**) HSF1 when trimerized and phosphorilated translocates in the nucleus where it binds HSEs DNA sequences to control heat shock proteins (**d**) In hepatocellular tumors, HSF1 overexpression correlates with c-Myc-dependent control of genes involved in tumorigenic pathways

The core of the mammalian Hippo pathway is a protein kinase cascade that consists of a pair of related serine/threonine kinases, mammalian STE20-like protein kinase 1 (MST1; and MST2). Other members of the Hippo pathway are large tumor suppressor 1 (LATS1) and LATS2 [47, 48]. Following upstream activation, the downstream Mst1/2 kinases, together with MOB1 cofactor and with the help of SAV1, phosphorylate and activate the LATS1/2 kinases. These proteins limit tissue growth by phosphorylation and inactivation of the homologous oncoproteins Yes associated protein (YAP) and transcriptional co-activator with PDZ-binding motif (TAZ) [49]. Phosphorylation of YAP and TAZ represses their activity promoting their cytoplasmic localization and ubiquitin-mediated degradation [50]. Unphosphorylated YAP/TAZ promotes tissue growth and cell viability translocating in the nucleus and forming complexes with transcription factors, consequently inducing the expression of targeted genes, by regulating the activity of different transcription factors, including TEADs and SMADs [51]. The nuclear localization of YAP was detected in several human cancers [52–54] where it induces cell proliferation and tissue growth. The cancer-associated signaling networks often engage crosstalk with the Hippo pathway at the level of the YAP and TAZ oncoproteins. An increased activity of YAP and TAZ was observed in high-grade metastatic breast cancer compared with low-grade non-metastatic breast cancer [55]. Noteworthy, it was shown a direct interaction between c-Myc and YAP–TEAD, at the transcriptional level, which integrates mitogenic and mechanical cues to provide multifactorial control of cell proliferation. YAP assists c-Myc-dependent transcription and cooperates in inducing cell cycle entry and cell proliferation both *in vitro* and *in vivo*. In a transgenic mouse model aimed to study the effects of c-Myc and YAP induction on liver growth, it was found that co-induction of c-Myc and YAP led to massive hepatomegaly, which accounted for the remarkably short disease-free survival of these mice [56]. Interestingly, these enlarged livers could be histologically classified as due to the presence of hepatocellular carcinomas (HCC), suggesting a direct involvement of Myc and YAP in the development of this tumor. In particular, c-Myc protein was reduced and the transcription of c-Myc was significantly inhibited when YAP was knocked-down, suggesting that YAP regulates c-Myc transcriptional activity. Furthermore, YAP regulates c-Myc via c-Abl, primarily at transcriptional level and, in liver cancer, c-Myc regulates YAP independent of transcription. Based on these data a regulation loop has been proposed, in which YAP drives c-Myc transcription via interaction with c-Abl, hence the up-regulation of c-Myc protects and enhances YAP protein expression [41]. Turato C. et al [57] showed another evidence of SerpinB3-dependent Yap-Myc interaction in liver cancer [57]. An indirect interaction between c-Myc and YAP/TAZ was demonstrated in mammary epithelial cells and in breast cancer, where c-Myc behaves as a potent repressor of YAP/TAZ function [58]. In oral squamous cells carcinoma (OSCC), YAP could regulate the expression of c-Myc since the knockdown of YAP inhibited the expression of c-Myc, while YAP overexpression showed opposite effects both at mRNA and protein levels. These data suggest that YAP could regulate c-Myc transcriptional activity and this led to sustained cell proliferation of the tumor [59]. In chronic myeloid leukemia (CML), where c-Myc is upregulated by BCR/ABL [60]. Li et al. [61] showed that knockdown of YAP down-regulates c-Myc both at protein and mRNA levels. Furthermore they found that both genetic and pharmacological inhibition of YAP markedly reduced the expression of c-Myc. In gastric tumor, Yap/Taz activation initiates gastric tumorigenesis *in vivo*. RNA-seq experiments identified c-Myc as a key downstream molecular target of Yap, which directly controls c-Myc at both transcriptional and post-transcriptional levels (Fig. 3a, b).

### HSF1 is a pivotal cofactor in c-Myc-driven tumorigenesis

Heat shock factor 1 (HSF1) is a stress inducible transcription factor that, in non-stressed status, is involved in a repressive complex comprising HSP-90 and HDAC6. When triggered, HSF1 becomes trimerized and phosphorylated, and then translocated into the nucleus, where it binds to conserved heat shock-responsive DNA elements (HSEs) to upregulate genes coding for heat shock proteins (HSPs) [62].Otherwise, it is now evident that HSF1 can bind only a subset of its potential HSEs *in vivo* indicating that, beyond the HSE, the local chromatin structure and interaction of different transcription factors may play an important role in transcriptional response to heat stress [63]. HSF1 has also been reported to influence numerous developmental events and cellular processes. Its mechanism of action involves the transcription of numerous genes encoding proteins others than HSPs, largely in a temperature-independent manner. In particular, the regulatory sequence of some HSF1 target genes does not contain a conserved HSE element [64]. In tumors, HSF1 primarily supports the survival of cells by increasing their stress tolerance. It also elevates the ability of cancer cells to resist various stress factors [43]. HSF1 can affect nodal points in oncogenic signaling by different mechanisms, such as transcriptional activation of signaling proteins, or regulation of mRNA translation or amplification of signaling pathways [65]. A growing number of studies have demonstrated that HSF1 is overexpressed in a series of solid tumors, and elevation of HSF1 expression is correlated with poor survival of tumor patients [66]. In esophageal cancer accumulated Myc directly potentiated HIF 1 transcription and then activated VEGF expression [67]. In HCC, it was found that HSF1 is necessary for sustaining the activity of the mTOR pathway and that its depletion strongly reinforces apoptosis in mouse embryonic fibroblasts overexpressing c-Myc. This evidence drives investigation on the functional interplay between HIF1 and c-Myc. It was found that HSF1 was strongly upregulated at mRNA and protein level in c-Myc-positive tumor samples. Downregulation of HSF1 in mouse livers and in human HCC cell lines correlates with low levels of c-Myc, as well as of c-Myc targets involved in *de novo* lipogenesis, mitochondrial biogenesis, polyamine metabolism and glycolysis. HIF1 depletion resulted also in reduced proliferation and increased apoptosis. Furthermore, the analysis of HIF1 and c-Myc correlation in HCC specimens showed that amplification of either molecules belonging to the HCC subgroup with poorer prognosis and a more aggressive phenotype. Altogether, these experimental evidences indicate that HSF1 is a pivotal c-Myc cofactor required for liver tumor tumorigenesis [68–70] (Fig. 3c, d).

## Conclusions

The revision of the latest discovered c-Myc cofactors strongly supports the hypothesis that c-Myc activates and represses selected target genes, with RNA amplification occurring only as a secondary phenomenon (Table 1). Physiological processes like cell size, energy metabolism, translation and nucleotide biosynthesis are controlled by various c-Myc regulated-genes that may thereby indirectly connect c-Myc activity to the general phenomenon of RNA amplification, associated with cell activation and transformation. This scenario is in contrast with the hypothesis where c-Myc is not seen as a specific transcription factor that is able to either activate or repress transcription of selected genes, but rather as a general amplifier with the ability to interact with all active regulatory elements, occupying them when expressed at high level. Furthermore the repression of c-Myc-controlled genes seem to be not due to supernumerary c-Myc molecules but by loss of RNA polymerase II from their promoters. Since several genes encoding RNA polymerase II subunit showed c-Myc-dependent expression, this is another proof of an indirect link between c-Myc and the global transcriptional activity [71]. The selected cofactors described here demonstrate that c-Myc needs to cooperate with specific molecules to exert the transcriptional control of selected genes in order to secondly influence a broad range of cellular functions, such as cell cycle entry, proliferation of tumor cells and cancer progression [72].

**Table 1:** Schematic view of activities and functions of c-Myc cofactors

Cofactors	Complex	Activity	Function
Che-1	-	Activator	Proliferation
INI1	Swi/Snf	Repressor	Trascription
BPTF	Nurf	Activator	Tumor initiation Tumor progression
YAP	Yap/Taz	Activator	Proliferation
			Tumorigenesis
HSF1	-	Activator	Tumorigenesis
			De Novo Lipogenesis
			Mitochondrial Biogenesis
			Polyamine metabolism
			Glycolysis

## Abbreviations

ALV: Avian leucosis virus
MAX: Myc-associated factor X
bHLH-LZ: Basic helix-loop-helix-leucine-zipper
HAT: Histone acetyl-transferase
HADC: Histone deacetylase
Miz-1: Myc-interacting Zn finger protein-1
MB: Medulloblastoma
TRRAP: Transcriptional histone acetyltransferase cofactor
CBP: CREB binding protein
SNIP1: Smad nuclear interacting protein 1
EGR1: Early growth response 1
pTEFb: Positive transcription elongation factor b
SP1: Specificity protein 1
Che-1/AATF: Anti-apoptotic transcription factor
NRAGE: Neurotrophin receptor-interacting MAGE homolog
MK2: MAPKAPK2
mTOR: Mammalian target of Rapamycin
BCP-ALL: B-cell precursor acute lymphoblastic leukemia
INI1: Integrase interactor 1
SWI/SNF5: Switching/sucrose non-fermenting
Co-IP: Co-immuneprecipitation
RBM38: RNA-binding protein 38
STAGA: SPT3-TAF (II) 31-GCN5L acetylase
Nurd: Nucleosome remodeling and deacetylase
BPTF: Bromodomain PHD transcription factor
Nurf: Nucleosome remodeling factor
MST: mammalian STE20 like protein kinase
LATS: Large tumor suppressor
MOB1: Mps one binder
SAV1: Salvador 1
YAP: Yes associated protein
TAZ: Transcriptional coactivator with PDZ-binding
TEAD: Transcriptional enhancer factor TEF1
SMAD: Small mother against decapentaplegic
OSCC: Oral squamous cells carcinoma
CML: Chronic myeloid leukemia
BCR/ABL: Breakpoint cluster region/Abelson
HSF1: Heat shock factor
HSP: Heat shock protein
HCC: Hepatocellular carcinoma
VEGF: Vascular endothelial growth factor
HIF: Hypoxia-inducible factor1

## References

[1] Beroukhim R, Mermel CH, Porter D, Wei G, Raychaudhuri S, Donovan J (2010). The landscape of somatic copy-number alteration across human cancers. Nature..

[2] McKeown MR, Bradner JE (2014). Therapeutic strategies to inhibit MYC. Cold Spring Harb Perspect Med..

[3] Hayward WS, Neel BG, Astrin SM (1981). Activation of a cellular onc gene by promoter insertion in ALV-induced lymphoid leukosis. Nature..

[4] Conacci-Sorrell M, McFerrin L, Eisenman RN (2014). An overview of MYC and its interactome. Cold Spring Harb Perspect Med..

[5] Adams JM, Harris AW, Pinkert CA, Corcoran LM, Alexander WS, Cory S (1985). The c-myc oncogene driven by immunoglobulin enhancers induces lymphoid malignancy in transgenic mice. Nature..

[6] Herbst A, Salghetti SE, Kim SY, Tansey WP (2004). Multiple cell-type-specific elements regulate Myc protein stability. Oncogene..

[7] Richart L, Carrillo-de Santa Pau E, Rio-Machin A, de Andres MP, Cigudosa JC, Lobo VJ (2016). BPTF is required for c-MYC transcriptional activity and in vivo tumorigenesis. Nat Commun..

[8] Eilers M, Eisenman RN (2008). Myc’s broad reach. Genes Dev..

[9] Hann SR (2014). MYC cofactors: molecular switches controlling diverse biological outcomes. Cold Spring Harb Perspect Med..

[10] Lin CY, Loven J, Rahl PB, Paranal RM, Burge CB, Bradner JE (2012). Transcriptional amplification in tumor cells with elevated c-Myc. Cell..

[11] Nie Z, Hu G, Wei G, Cui K, Yamane A, Resch W (2012). c-Myc is a universal amplifier of expressed genes in lymphocytes and embryonic stem cells. Cell..

[12] Yap CS, Peterson AL, Castellani G, Sedivy JM, Neretti N (2011). Kinetic profiling of the c-Myc transcriptome and bioinformatic analysis of repressed gene promoters. Cell Cycle..

[13] Mao DY, Watson JD, Yan PS, Barsyte-Lovejoy D, Khosravi F, Wong WW (2003). Analysis of Myc bound loci identified by CpG island arrays shows that Max is essential for Myc-dependent repression. Curr Biol..

[14] Kress TR, Sabo A, Amati B (2015). MYC: connecting selective transcriptional control to global RNA production. Nat Rev Cancer..

[15] Sabo A, Kress TR, Pelizzola M, de Pretis S, Gorski MM, Tesi A (2014). Selective transcriptional regulation by Myc in cellular growth control and lymphomagenesis. Nature..

[16] Peukert K, Staller P, Schneider A, Carmichael G, Hanel F, Eilers M (1997). An alternative pathway for gene regulation by Myc. EMBO J..

[17] Vo BT, Wolf E, Kawauchi D, Gebhardt A, Rehg JE, Finkelstein D (2016). The Interaction of Myc with Miz1 Defines Medulloblastoma Subgroup Identity. Cancer Cell..

[18] Dugan KA, Wood MA, Cole MD (2002). TIP49, but not TRRAP, modulates c-Myc and E2F1 dependent apoptosis. Oncogene..

[19] Vervoorts J, Luscher-Firzlaff JM, Rottmann S, Lilischkis R, Walsemann G, Dohmann K (2003). Stimulation of c-MYC transcriptional activity and acetylation by recruitment of the cofactor CBP. EMBO Rep..

[20] Fujii M, Lyakh LA, Bracken CP, Fukuoka J, Hayakawa M, Tsukiyama T (2006). SNIP1 is a candidate modifier of the transcriptional activity of c-Myc on E box-dependent target genes. Mol Cell..

[21] Boone DN, Qi Y, Li Z, Hann SR (2011). Egr1 mediates p53-independent c-Myc-induced apoptosis via a noncanonical ARF-dependent transcriptional mechanism. Proc Natl Acad Sci U S A..

[22] Gargano B, Amente S, Majello B, Lania L (2007). P-TEFb is a crucial co-factor for Myc transactivation. Cell Cycle..

[23] Zhang Y, Chen HX, Zhou SY, Wang SX, Zheng K, Xu DD (2015). Sp1 and c-Myc modulate drug resistance of leukemia stem cells by regulating survivin expression through the ERK-MSK MAPK signaling pathway. Mol Cancer..

[24] Bruno T, De Angelis R, De Nicola F, Barbato C, Di Padova M, Corbi N (2002). Che-1 affects cell growth by interfering with the recruitment of HDAC1 by Rb. Cancer Cell..

[25] Sorino C, Bruno T, Desantis A, Di Certo MG, Iezzi S, De Nicola F (2013). Centrosomal Che-1 protein is involved in the regulation of mitosis and DNA damage response by mediating pericentrin (PCNT)-dependent Chk1 protein localization. J Biol Chem..

[26] Di Certo MG, Corbi N, Bruno T, Iezzi S, De Nicola F, Desantis A (2007). NRAGE associates with the anti-apoptotic factor Che-1 and regulates its degradation to induce cell death. J Cell Sci..

[27] Hopker K, Hagmann H, Khurshid S, Chen S, Hasskamp P, Seeger-Nukpezah T (2012). AATF/Che-1 acts as a phosphorylation-dependent molecular modulator to repress p53-driven apoptosis. EMBO J..

[28] De Nicola F, Bruno T, Iezzi S, Di Padova M, Floridi A, Passananti C (2007). The prolyl isomerase Pin1 affects Che-1 stability in response to apoptotic DNA damage. J Biol Chem..

[29] Bruno T, Desantis A, Bossi G, Di Agostino S, Sorino C, De Nicola F (2010). Che-1 promotes tumor cell survival by sustaining mutant p53 transcription and inhibiting DNA damage response activation. Cancer Cell..

[30] Bruno T, Valerio M, Casadei L, De Nicola F, Goeman F, Pallocca M (2017). Che-1 sustains hypoxic response of colorectal cancer cells by affecting Hif-1alpha stabilization. J Exp Clin Cancer Res..

[31] Folgiero V, Sorino C, Pallocca M, De Nicola F, Goeman F, Bertaina V (2018). Che-1 is targeted by c-Myc to sustain proliferation in pre-B-cell acute lymphoblastic leukemia. EMBO Rep..

[32] Kadoch C, Hargreaves DC, Hodges C, Elias L, Ho L, Ranish J (2013). Proteomic and bioinformatic analysis of mammalian SWI/SNF complexes identifies extensive roles in human malignancy. Nat Genet..

[33] Jackson EM, Sievert AJ, Gai X, Hakonarson H, Judkins AR, Tooke L (2009). Genomic analysis using high-density single nucleotide polymorphism-based oligonucleotide arrays and multiplex ligation-dependent probe amplification provides a comprehensive analysis of INI1/SMARCB1 in malignant rhabdoid tumors. Clin Cancer Res..

[34] Cheng SW, Davies KP, Yung E, Beltran RJ, Yu J, Kalpana GV (1999). c-MYC interacts with INI1/hSNF5 and requires the SWI/SNF complex for transactivation function. Nat Genet..

[35] Stojanova A, Tu WB, Ponzielli R, Kotlyar M, Chan PK, Boutros PC (2016). MYC interaction with the tumor suppressive SWI/SNF complex member INI1 regulates transcription and cellular transformation. Cell Cycle..

[36] Wu DY, Tkachuck DC, Roberson RS, Schubach WH (2002). The human SNF5/INI1 protein facilitates the function of the growth arrest and DNA damage-inducible protein (GADD34) and modulates GADD34-bound protein phosphatase-1 activity. J Biol Chem..

[37] DeCristofaro MF, Betz BL, Wang W, Weissman BE (1999). Alteration of hSNF5/INI1/BAF47 detected in rhabdoid cell lines and primary rhabdomyosarcomas but not Wilms’ tumors. Oncogene..

[38] Li XX, Shi L, Zhou XJ, Wu J, Xia TS, Zhou WB (2017). The role of c-Myc-RBM38 loop in the growth suppression in breast cancer. J Exp Clin Cancer Res..

[39] Dingar D, Kalkat M, Chan PK, Srikumar T, Bailey SD, Tu WB (2015). BioID identifies novel c-MYC interacting partners in cultured cells and xenograft tumors. J Proteomics..

[40] Zhang ZK, Davies KP, Allen J, Zhu L, Pestell RG, Zagzag D (2002). Cell cycle arrest and repression of cyclin D1 transcription by INI1/hSNF5. Mol Cell Biol..

[41] Xiao W, Wang J, Ou C, Zhang Y, Ma L, Weng W (2013). Mutual interaction between YAP and c-Myc is critical for carcinogenesis in liver cancer. Biochem Biophys Res Commun..

[42] Landry J, Sharov AA, Piao Y, Sharova LV, Xiao H, Southon E (2008). Essential role of chromatin remodeling protein Bptf in early mouse embryos and embryonic stem cells. PLoS Genet..

[43] Dai M, Lu JJ, Guo W, Yu W, Wang Q, Tang R (2015). BPTF promotes tumor growth and predicts poor prognosis in lung adenocarcinomas. Oncotarget..

[44] Mayes K, Elsayed Z, Alhazmi A, Waters M, Alkhatib SG, Roberts M (2017). BPTF inhibits NK cell activity and the abundance of natural cytotoxicity receptor co-ligands. Oncotarget..

[45] Mayes K, Alkhatib SG, Peterson K, Alhazmi A, Song C, Chan V (2016). BPTF Depletion Enhances T-cell-Mediated Antitumor Immunity. Cancer Res..

[46] Vicent GP, Nacht AS, Font-Mateu J, Castellano G, Gaveglia L, Ballare C (2011). Four enzymes cooperate to displace histone H1 during the first minute of hormonal gene activation. Genes Dev..

[47] Udan RS, Kango-Singh M, Nolo R, Tao C, Halder G (2003). Hippo promotes proliferation arrest and apoptosis in the Salvador/Warts pathway. Nat Cell Biol..

[48] Wu S, Huang J, Dong J, Pan D (2003). hippo encodes a Ste-20 family protein kinase that restricts cell proliferation and promotes apoptosis in conjunction with salvador and warts. Cell..

[49] Huang J, Wu S, Barrera J, Matthews K, Pan D (2005). The Hippo signaling pathway coordinately regulates cell proliferation and apoptosis by inactivating Yorkie, the Drosophila Homolog of YAP. Cell..

[50] Zhao B, Wei X, Li W, Udan RS, Yang Q, Kim J (2007). Inactivation of YAP oncoprotein by the Hippo pathway is involved in cell contact inhibition and tissue growth control. Genes Dev..

[51] Hong W, Guan KL (2012). The YAP and TAZ transcription co-activators: key downstream effectors of the mammalian Hippo pathway. Semin Cell Dev Biol..

[52] Zhang X, George J, Deb S, Degoutin JL, Takano EA, Fox SB (2011). The Hippo pathway transcriptional co-activator, YAP, is an ovarian cancer oncogene. Oncogene..

[53] Xu MZ, Yao TJ, Lee NP, Ng IO, Chan YT, Zender L (2009). Yes-associated protein is an independent prognostic marker in hepatocellular carcinoma. Cancer..

[54] Wang Y, Dong Q, Zhang Q, Li Z, Wang E, Qiu X (2010). Overexpression of yes-associated protein contributes to progression and poor prognosis of non-small-cell lung cancer. Cancer Sci..

[55] Cordenonsi M, Zanconato F, Azzolin L, Forcato M, Rosato A, Frasson C (2011). The Hippo transducer TAZ confers cancer stem cell-related traits on breast cancer cells. Cell..

[56] Croci O, De Fazio S, Biagioni F, Donato E, Caganova M, Curti L (2017). Transcriptional integration of mitogenic and mechanical signals by Myc and YAP. Genes Dev..

[57] Turato C, Cannito S, Simonato D, Villano G, Morello E, Terrin L (2015). SerpinB3 and Yap Interplay Increases Myc Oncogenic Activity. Sci Rep..

[58] von Eyss B, Jaenicke LA, Kortlever RM, Royla N, Wiese KE, Letschert S (2015). A MYC-Driven Change in Mitochondrial Dynamics Limits YAP/TAZ Function in Mammary Epithelial Cells and Breast Cancer. Cancer Cell..

[59] Chen X, Gu W, Wang Q, Fu X, Wang Y, Xu X (2017). C-MYC and BCL-2 mediate YAP-regulated tumorigenesis in OSCC. Oncotarget..

[60] Gomez-Casares MT, Vaque JP, Lemes A, Molero T, Delgado MD, Leon J (2004). C-myc expression in cell lines derived from chronic myeloid leukemia. Haematologica..

[61] Li H, Huang Z, Gao M, Huang N, Luo Z, Shen H (2016). Inhibition of YAP suppresses CML cell proliferation and enhances efficacy of imatinib in vitro and in vivo. J Exp Clin Cancer Res..

[62] Barna J, Csermely P, Vellai T (2018). Roles of heat shock factor 1 beyond the heat shock response. Cell Mol Life Sci..

[63] Guertin MJ, Lis JT (2010). Chromatin landscape dictates HSF binding to target DNA elements. PLoS Genet..

[64] Stephanou A, Latchman DS (2011). Transcriptional modulation of heat-shock protein gene expression. Biochem Res Int..

[65] Home T, Jensen RA, Rao R (2015). Heat shock factor 1 in protein homeostasis and oncogenic signal integration. Cancer Res..

[66] Wan T, Shao J, Hu B, Liu G, Luo P, Zhou Y (2018). Prognostic role of HSF1 overexpression in solid tumors: a pooled analysis of 3,159 patients. Onco Targets Ther..

[67] Ma S, Lu CC, Yang LY, Wang JJ, Wang BS, Cai HQ (2018). ANXA2 promotes esophageal cancer progression by activating MYC-HIF1A-VEGF axis. J Exp Clin Cancer Res..

[68] Cigliano A, Pilo MG, Li L, Latte G, Szydlowska M, Simile MM (2017). Deregulated c-Myc requires a functional HSF1 for experimental and human hepatocarcinogenesis. Oncotarget..

[69] Li J, Liu Q, Liu Z, Xia Q, Zhang Z, Zhang R (2018). KPNA2 promotes metabolic reprogramming in glioblastomas by regulation of c-myc. J Exp Clin Cancer Res..

[70] Li SG, Shi QW, Yuan LY, Qin LP, Wang Y, Miao YQ (2018). C-Myc-dependent repression of two oncogenic miRNA clusters contributes to triptolide-induced cell death in hepatocellular carcinoma cells. J Exp Clin Cancer Res..

[71] Sabo A, Amati B (2018). BRD4 and MYC-clarifying regulatory specificity. Science..

[72] Yoshida GJ (2018). Emerging roles of Myc in stem cell biology and novel tumor therapies. J Exp Clin Cancer Res..

